# Certain Hermite-Hadamard type inequalities via generalized *k*-fractional integrals

**DOI:** 10.1186/s13660-017-1318-y

**Published:** 2017-03-04

**Authors:** Praveen Agarwal, Mohamed Jleli, Muharrem Tomar

**Affiliations:** 1 Department of Mathematics, Anand International College of Engineering, Jaipur, 303012 India; 2 Department of Mathematics, King Saud University, Riyadh, 11451 Kingdom of Saudi Arabia; 3 Department of Mathematics, Faculty of Arts and Sciences, Ordu University, Ordu, 52200 Turkey

**Keywords:** 26A33, 26A51, 26D15, Hermite-Hadamard inequality, generalized *k*-fractional integral, $(k, s)$-fractional integral, $(k, s)$-Riemann-Liouville fractional integral

## Abstract

Some Hermite-Hadamard type inequalities for generalized *k*-fractional integrals (which are also named $(k,s)$-Riemann-Liouville fractional integrals) are obtained for a fractional integral, and an important identity is established. Also, by using the obtained identity, we get a Hermite-Hadamard type inequality.

## Introduction

Let $f:I\subseteq\mathbb{R}\rightarrow\mathbb{R}$ be a convex function defined on the interval *I* of real numbers and $a,b\in I$ with $a< b$. The following inequality

1.1 $$ f \biggl(\frac{a+b}{2} \biggr)\leq\frac{1}{b-a} \int_{a}^{b}f(x)\,dx\leq\frac {f(a)+f(b)}{2} $$

holds. This double inequality is known in the literature as a Hermite-Hadamard integral inequality for convex functions [1].

Sarikaya et al. established the following results for Riemann-Liouville fractional integrals.

### Theorem 1.1

see Theorem 2 in [2]

*Let* $f:[a,b]\rightarrow\mathbb{R}$ *be a positive function with* $0\leq a< b$ *and* $f\in L_{1}[a,b]$. *If* *f* *is a convex function on* $[a,b]$, *then the following inequality for fractional integrals holds*:

1.2 $$ f \biggl(\frac{a+b}{2} \biggr)\leq\frac{\Gamma(1+\alpha)}{2(b-a)^{\alpha}} \bigl[J_{a^{+}}^{\alpha}f(b)+J_{b^{-}}^{\alpha}f(a) \bigr]\leq\frac{f(a)+f(b)}{2} $$

*with* $\alpha>0$, *where the symbols* $J_{a^{+}}^{\alpha}$ *and* $J_{b^{-}}^{\alpha}$ *denote the left*-*sided and right*-*sided Riemann*-*Liouville fractional integrals of the order* $\alpha\in\mathbb{R}^{+}$ *that are defined by*

$$J_{a^{+}}^{\alpha}f(x)=\frac{1}{\Gamma(\alpha)} \int_{a}^{x} f(t) (x-t)^{\alpha -1}\,dt \quad (0 \leq a < x\leq b) $$

*and*

$$J_{b^{-}}^{\alpha}f(x)=\frac{1}{\Gamma(\alpha)} \int_{x}^{b} f(t) (t-x)^{\alpha -1}\,dt\quad (0\leq a \leq x< b) $$

*respectively*. *Here* $\Gamma(\cdot)$ *denotes the classical gamma function* [3], *Chapter *6.

### Theorem 1.2

see Theorem 3 in [2]

*Let* $f:[a,b]\rightarrow\mathbb {R}$ *be a differentiable mapping on* $(a,b)$ *with* $a< b$. *If* $f'\in L[a,b]$, *then the following inequality for Riemann*-*Liouville fractional integrals holds*:

1.3 $$\begin{aligned}& \biggl\vert \frac{f(a)+f(b)}{2}-\frac{\Gamma(\alpha+1)}{2(b-a)^{\alpha}} \bigl[J_{a^{+}}^{\alpha}f(b)+J_{b^{-}}^{\alpha}f(a) \bigr]\biggr\vert \\& \quad \leq\frac{b-a}{2(\alpha+1)} \biggl(1-\frac{1}{2^{\alpha}} \biggr) \bigl(\bigl\vert f'(a)\bigr\vert +\bigl\vert f'(b)\bigr\vert \bigr) \end{aligned}$$

*with* $\alpha>0$.

The Pochhammer *k*-symbol $(x)_{n,k}$ and the *k*-gamma function $\Gamma _{k}$ are defined as follows (see [4]):

1.4 $$ (x)_{n,k}:=x(x+k) (x+2k)\cdots \bigl(x+(n-1)k \bigr) \quad (n \in\mathbb {N}; k >0 ) $$

and

1.5 $$ \Gamma_{k}(x):= \lim_{n \rightarrow\infty} \frac{n! k^{n} (nk)^{\frac {x}{k}-1}}{(x)_{n,k}}\quad \bigl(k >0; x \in\mathbb{C}\setminus k \mathbb{Z}_{0}^{-} \bigr), $$

where $k \mathbb{Z}_{0}^{-}:= \{kn : n \in\mathbb{Z}_{0}^{-} \}$. It is noted that the case $k=1$ of (1.4) and (1.5) reduces to the familiar Pochhammer symbol $(x)_{n}$ and the gamma function Γ. The function $\Gamma_{k}$ is given by the following integral:

1.6 $$ \Gamma_{k}(x)= \int_{0}^{\infty}t^{x-1} e^{-\frac{t^{k}}{k}} \,dt \quad \bigl(\Re(x)>0\bigr). $$

The function $\Gamma_{k}$ defined on $\mathbb{R}^{+}$ is characterized by the following three properties: (i) $\Gamma_{k}(x+k)=x \Gamma_{k}(x)$; (ii) $\Gamma_{k}(k)=1$; (iii) $\Gamma _{k}(x)$ is logarithmically convex. It is easy to see that

1.7 $$ \Gamma_{k}(x)= k^{\frac{x}{k}-1} \Gamma \biggl( \frac{x}{k} \biggr)\quad \bigl(\Re (x)>0; k >0 \bigr). $$

We want to recall the preliminaries and notations of some well-known fractional integral operators that will be used to obtain some remarks and corollaries.

The $(k,s)$-Riemann-Liouville fractional integral operator $_{k}^{s}\mathcal{J}_{a}^{\alpha}$ of order $\alpha>0$ for a real-valued continuous function $f(t)$ is defined as (see [5], p.79, 2.1. Definition):

1.8 $$ {}_{k}^{s}\mathcal{J}_{a}^{\alpha}f(x)=\frac{(s+1)^{1-\frac{\alpha }{k}}}{k\Gamma_{k}(\alpha)} \int_{a}^{x}\bigl(x^{s+1}-t^{s+1} \bigr)^{\frac{\alpha }{k}-1}t^{s}f(t)\,dt, $$

where $k>0$, $\beta>0$ and $s \in\mathbb{R}\setminus\{-1\}$.

The most important feature of $(k,s)$-fractional integrals is that they generalize some types of fractional integrals (Riemann-Liouville fractional integral, *k*-Riemann-Liouville fractional integral, generalized fractional integral and Hadamard fractional integral). These important special cases of the integral operator ${}_{k}^{s}\mathcal {J}_{a}^{\alpha}$ are mentioned below.

1. For $k=1$, the operator in (1.8) yields the following generalized fractional integrals defined by Katugompola in [6]:
  1.9 $$ {}_{a}^{r}\mathcal{J}_{t}^{\alpha}f(x)=\frac{(r+1)^{1-\alpha}}{\Gamma(\alpha )} \int_{a}^{x}\bigl(x^{r+1}-t^{r+1} \bigr)^{\alpha-1}t^{r}f(t)\,dt. $$
2. Firstly by taking $k=1$, after that by taking limit $r\rightarrow{-1^{+}}$ and using L’Hôpital’s rule, the operator in (1.8) leads to the Hadamard fractional integral operator [1, 7]. That is,
  1.10 $$\begin{aligned} \lim_{r \rightarrow-1^{+}} {}_{a}^{r} \mathcal{J}_{t}^{\alpha}f(x) =&\lim_{r \rightarrow-1^{+}} \frac{(r+1)^{1-\alpha}}{\Gamma(\alpha)} \int_{a}^{x}\frac{f(t)t^{r}}{(x^{r+1}-t^{r+1})^{1-\alpha}}\,dt \\ =&\frac{1}{\Gamma(\alpha)} \int_{a}^{x}\lim_{r \rightarrow -1^{+}}f(t)t^{r} \biggl(\frac{r+1}{x^{r+1}-t^{r+1}} \biggr)^{1-\alpha}\,dt \\ =& \frac{1}{\Gamma(\alpha)} \int_{a}^{x}f(t)\lim_{r\rightarrow-1^{+}} \biggl( \frac{r+1}{x^{r+1}-t^{r+1}} \biggr)^{1-\alpha}\frac {dt}{t} \\ =&\frac{1}{\Gamma(\alpha)} \int_{a}^{x}f(t) \biggl(\lim_{r \rightarrow -1^{+}} \frac{r+1}{x^{r+1}-t^{r+1}} \biggr)^{1-\alpha}\frac{dt}{t} \\ =&\frac{1}{\Gamma(\alpha)} \int_{a}^{x} \biggl(\log\frac{x}{t} \biggr)f(t)\frac {dt}{t} \\ =& _{H}\mathcal{J}^{\alpha}\bigl[f(t)\bigr] \end{aligned}$$
  (see [8], p.569, eq. (3.13)).
3. If we take $s=0$ in (1.8), operator (1.8), reduces to the *k*-Riemann-Liouville fractional integral operator, which has been firstly defined by Mubeen and Habibullah in [9]. This relation is as follows:
  1.11 $$ \mathcal{J}_{a,k}^{\alpha}\, f(x)=\frac{1}{k\Gamma_{k}(\alpha)} \int _{a}^{x}(x-t)^{\frac{\alpha}{k}-1}f(t)\,dt. $$
4. Again, taking $s=0$ and $k=1$, operator (1.8) gives us the Riemann-Liouville fractional integration operator
  1.12 $$ J_{a^{+}}^{\alpha}f(x)=\frac{1}{\Gamma(\alpha)} \int_{a}^{x}(x-t)^{\alpha-1}f(t)\,dt. $$

In recent years, these fractional operators have been studied and used to extend especially Grüss, Chebychev-Grüss and Pólya-Szegö type inequalities. For more details, one may refer to the recent works and books [7, 10–21].

## Main results

Let $f:I^{\circ}\rightarrow\mathbb{R}$ be a given function, where $a,b\in I^{\circ}$ and $0< a< b<\infty$. We suppose that $f\in L_{\infty}(a,b)$ such that ${}_{k}^{s}J_{a^{+}}^{\alpha}f ( x )$ and ${}_{k}^{s}J_{b^{-}}^{\alpha}f ( x )$ are well defined. We define functions

$$\tilde{f}(x):=f(a+b-x),\quad x\in[a,b] $$

and

$$F(x):=f(x)+\tilde{f}(x),\quad x\in[a,b]. $$

Hermite-Hadamard’s inequality for convex functions can be represented in a $(k,s)$-fractional integral form as follows by using the change of variables $u=\frac{t-a}{x-a}$; we have from (1.8)

2.1 $$\begin{aligned} {}_{k}^{s}\mathcal{J}_{a}^{\alpha}f(x) =&(x-a) \frac{(s+1)^{1-\frac{\alpha }{k}}}{k\Gamma_{k}(\alpha)} \int_{0}^{1}\frac {(ux+(1-u)a)^{s}}{((ux+(1-u)a)^{s+1}-t^{s+1})^{\frac{\alpha }{k}-1}} \\ &{}\times f\bigl(ux+(1-u)a\bigr) \,ds, \end{aligned}$$

where $x>a$.

### Theorem 2.1

*Let* $\alpha,k>0$ *and* $s\in\mathbb{R}\setminus\{-1\}$. *If* *f* *is a convex function on* $[a,b]$, *then we have*

2.2 $$\begin{aligned} f \biggl(\frac{a+b}{2} \biggr) \leq&\frac{(s+1)^{\frac{\alpha}{k}}\Gamma_{k}(\alpha +k)}{4(b^{s+1}-a^{s+1})^{\frac{\alpha}{k}}} \bigl[ {}_{k}^{s}J_{a^{+}}^{\alpha}F(b)+ {}_{k}^{s}J_{b^{-}}^{\alpha}F(a) \bigr] \\ \leq&\frac{f(a)+f(b)}{2}. \end{aligned}$$

### Proof

For $u\in[0,1]$, let $\xi=au+(1-u)b $ and $\eta=(1-u)a+bu$. Using the convexity of *f*, we get

$$ f \biggl(\frac{a+b}{2} \biggr)=f \biggl(\frac{\xi+\eta}{2} \biggr) \leq \frac{1}{2}f(\xi)+\frac{1}{2}f(\eta). $$

That is,

2.3 $$ f \biggl(\frac{a+b}{2} \biggr) \leq\frac{1}{2}f \bigl(au+(1-u)b \bigr)+\frac{1}{2}f \bigl((1-u)a+bu \bigr). $$

Now, multiplying both sides of (2.3) by

$$(b-a)\frac{(s+1)^{1-\frac{\alpha}{k}}}{k\Gamma_{k}(\alpha)} \frac{(ub+(1-u)a)^{s}}{ [b^{s+1}-(ub+(1-u)a)^{s+1} ]^{1-\frac {\alpha}{k}}} $$

and integrating over $(0,1)$ with respect to *u*, we get

$$\begin{aligned}& (b-a)\frac{(s+1)^{1-\frac{\alpha}{k}}}{k\Gamma_{k}(\alpha)}f \biggl(\frac {a+b}{2} \biggr) \int_{0}^{1}\frac{(ub+(1-u)a)^{s} \,du}{ [b^{s+1}-(ub+(1-u)a)^{s+1} ]^{1-\frac{\alpha}{k}}} \\& \quad \leq\frac{1}{2}(b-a)\frac{(s+1)^{1-\frac{\alpha}{k}}}{k\Gamma_{k}(\alpha)} \int_{0}^{1}\frac{(ub+(1-u)a)^{s}f(au+(1-u)b)\,du }{ [b^{s+1}-(ub+(1-u)a)^{s+1} ]^{1-\frac{\alpha}{k}}} \\& \qquad {}+\frac{1}{2}(b-a)\frac{(s+1)^{1-\frac{\alpha}{k}}}{k\Gamma_{k}(\alpha)} \int_{0}^{1}\frac{(ub+(1-u)a)^{s}f((1-u)a+bu)\,du }{ [b^{s+1}-(ub+(1-u)a)^{s+1} ]^{1-\frac{\alpha}{k}}}. \end{aligned}$$

Note that we have

$$\int_{0}^{1}\frac{(ub+(1-u)a)^{s} \,du}{ [b^{s+1}-(ub+(1-u)a)^{s+1} ]^{1-\frac{\alpha}{k}}} =\frac{k (b^{s+1}-a^{s+1} )^{\frac{\alpha}{k}}}{\alpha(s+1)(b-a)}. $$

Using the identity

$$\tilde{f}\bigl((1-u)a+bu\bigr)=f\bigl(au+(1-u)b\bigr), $$

and from (2.1), we obtain

$$(b-a)\frac{(s+1)^{1-\frac{\alpha}{k}}}{k\Gamma_{k}(\alpha)} \int_{0}^{1}\frac{(ub+(1-u)a)^{s} f(au+(1-u)b) \,du}{ [b^{s+1}-(ub+(1-u)a)^{s+1} ]^{1-\frac{\alpha}{k}}} = {}_{k}^{s}J_{a^{+}}^{\alpha}\tilde{f}(b) $$

and

$$(b-a)\frac{(s+1)^{1-\frac{\alpha}{k}}}{k\Gamma_{k}(\alpha)} \int_{0}^{1}\frac{(ub+(1-u)a)^{s} f((1-u)a+bu) \,du}{ [b^{s+1}-(ub+(1-u)a)^{s+1} ]^{1-\frac{\alpha}{k}}} = {}_{k}^{s}J_{a^{+}}^{\alpha}f(b). $$

Accordingly, we have

2.4 $$ \frac{(b^{s+1}-a^{s+1})^{\frac{\alpha}{k}}}{(s+1)^{\frac{\alpha }{k}}\Gamma_{k}(\alpha+k)}f \biggl(\frac{a+b}{2} \biggr) \leq \frac{ {}_{k}^{s}J_{a^{+}}^{\alpha}F(b)}{2}. $$

Similarly, multiplying both sides of (2.3) by

$$(b-a)\frac{(s+1)^{1-\frac{\alpha}{k}}}{k\Gamma_{k}(\alpha)} \frac{(ub+(1-u)a)^{s}}{ [(bu+(1-u)a)^{s+1}-a^{s+1} ]^{1-\frac {\alpha}{k}}}, $$

integrating over $(0,1)$ with respect to *u*, and from (2.1), we also get

2.5 $$ \frac{(b^{s+1}-a^{s+1})^{\frac{\alpha}{k}}}{(s+1)^{\frac{\alpha }{k}}\Gamma_{k}(\alpha+k)}f \biggl(\frac{a+b}{2} \biggr) \leq \frac{ {}_{k}^{s}J_{b^{-}}^{\alpha}F(a)}{2}. $$

By adding inequalities (2.4) and (2.5), we get

$$ f \biggl(\frac{a+b}{2} \biggr) \leq\frac{(s+1)^{\frac{\alpha}{k}}\Gamma_{k}(\alpha +k)}{4(b^{s+1}-a^{s+1})^{\frac{\alpha}{k}}} \bigl[ {}_{k}^{s}J_{a^{+}}^{\alpha}F(b)+ {}_{k}^{s}J_{b^{-}}^{\alpha }F(a) \bigr], $$

which is the left-hand side of inequality (2.2).

Since *f* is convex, for $u\in[0,1]$, we have

2.6 $$ f \bigl(au+(1-u)b \bigr)+f \bigl((1-u)a+bu \bigr)\leq f(a)+f(b). $$

Multiplying both sides of (2.6) by

$$(b-a)\frac{(s+1)^{1-\frac{\alpha}{k}}}{k\Gamma_{k}(\alpha)} \frac{(ub+(1-u)a)^{s}}{ [b^{s+1}-(ub+(1-u)a)^{s+1} ]^{1-\frac {\alpha}{k}}} $$

and integrating over $(0,1)$ with respect to *u*, we get

$$\begin{aligned} \begin{aligned} &(b-a)\frac{(s+1)^{1-\frac{\alpha}{k}}}{k\Gamma_{k}(\alpha)} \int_{0}^{1}\frac{(ub+(1-u)a)^{s}f (au+(1-u)b )\,du}{ [b^{s+1}-(ub+(1-u)a)^{s+1} ]^{1-\frac{\alpha}{k}}} \\ &\qquad {}+(b-a)\frac{(s+1)^{1-\frac{\alpha}{k}}}{k\Gamma_{k}(\alpha)} \int_{0}^{1} \frac{(ub+(1-u)a)^{s}f ((1-u)a+bu )\,du}{ [b^{s+1}-(ub+(1-u)a)^{s+1} ]^{1-\frac{\alpha}{k}}} \\ &\quad \leq (b-a)\frac{(s+1)^{1-\frac{\alpha}{k}}}{k\Gamma_{k}(\alpha)} \bigl[f(a)+f(b) \bigr] \int_{0}^{1}\frac{(ub+(1-u)a)^{s} \,du}{ [b^{s+1}-(ub+(1-u)a)^{s+1} ]^{1-\frac{\alpha}{k}}}. \end{aligned} \end{aligned}$$

That is,

2.7 $$ {}_{k}^{s}J_{a^{+}}^{\alpha}F(b) \leq\frac{ (b^{s+1}-a^{s+1} )^{\frac{\alpha}{k}}}{(s+1)^{\frac {\alpha}{k}}\Gamma_{k}(\alpha+k)} \bigl[f(a)+f(b) \bigr]. $$

Similarly, multiplying both sides of (2.6) by

$$(b-a)\frac{(s+1)^{1-\frac{\alpha}{k}}}{k\Gamma_{k}(\alpha)} \frac{(ub+(1-u)a)^{s}}{ [(ub+(1-u)a)^{s+1}-a^{s+1} ]^{1-\frac {\alpha}{k}}} $$

and integrating over $(0,1)$ with respect to *u*, we also get

2.8 $$ {}_{k}^{s}J_{b^{-}}^{\alpha}F(a) \leq\frac{ (b^{s+1}-a^{s+1} )^{\frac{\alpha}{k}}}{(s+1)^{\frac {\alpha}{k}}\Gamma_{k}(\alpha+k)} \bigl[f(a)+f(b) \bigr]. $$

Adding inequalities (2.7) and (2.8), we obtain

$$ \frac{(s+1)^{\frac{\alpha}{k}}\Gamma_{k}(\alpha +k)}{4(b^{s+1}-a^{s+1})^{\frac{\alpha}{k}}} \bigl[ {}_{k}^{s}J_{a^{+}}^{\alpha}F(b)+ {}_{k}^{s}J_{b^{-}}^{\alpha }F(a) \bigr]\leq \frac{f(a)+f(b)}{2}, $$

which is the right-hand side of inequality (2.2). So the proof is complete. □

We want to give the following function that we will use later: For $\alpha,k>0$ and $s\in\mathbb{R}\setminus\{-1\}$, let $\nabla _{\alpha,s}:[0,1]\rightarrow\mathbb{R}$ be the function defined by

$$\begin{aligned} \nabla_{\alpha,s}(t): =& \bigl(\bigl(ta+(1-t)b\bigr)^{s+1}-a^{s+1} \bigr)^{\frac {\alpha}{k}} - \bigl(\bigl(bt+(1-t)a\bigr)^{s+1}-a^{s+1} \bigr)^{\frac{\alpha}{k}} \\ &{}+ \bigl(b^{s+1}-\bigl(tb+(1-t)a\bigr)^{s+1} \bigr)^{\frac{\alpha}{k}} - \bigl(b^{s+1}-\bigl(ta+(1-t)b \bigr)^{s+1} \bigr)^{\frac{\alpha}{k}}. \end{aligned}$$

In order to prove our main result, we need the following identity.

### Lemma 2.1

*Let* $\alpha,k>0$ *and* $s\in\mathbb{R}I^{\circ}$. *If* *f* *is a differentiable function on* $I^{\circ}$ *such that* $f'\in L[a,b]$ *with* $a< b$, *then we have the following identity*:

2.9 $$\begin{aligned}& \frac{f(a)+f(b)}{2}-\frac{(s+1)^{\frac{\alpha}{k}}\Gamma_{k}(\alpha +k)}{4(b^{s+1}-a^{s+1})^{\frac{\alpha}{k}}} \bigl[ {}_{k}^{s}J_{a^{+}}^{\alpha}F(b)+ {}_{k}^{s}J_{b^{-}}^{\alpha }F(a) \bigr] \\& \quad =\frac{(b-a)}{4(b^{s+1}-a^{s+1})^{\frac{\alpha}{k}}} \int_{0}^{1}\nabla_{\alpha ,s}(t)f' \bigl(ta+(1-t)b\bigr)\,dt. \end{aligned}$$

### Proof

Using integration by parts, we obtain

2.10 $$\begin{aligned} {}_{k}^{s}J_{a^{+}}^{\alpha}F(b) =&\frac{ (b^{s+1}-a^{s+1} )^{\frac{\alpha}{k}}}{(s+1)^{\frac {\alpha}{k}}\Gamma_{k}(\alpha+k)}F(a) +\frac{(b-a)}{(s+1)^{\frac{\alpha}{k}}\Gamma_{k}(\alpha+k)} \\ &{}\times \int_{0}^{1} \bigl[ \bigl(b^{s+1}- \bigl(bu+(1-u)a\bigr)^{s+1} \bigr) \bigr]^{\frac{\alpha}{k}}F'\bigl(bu+(1-u)a\bigr)\,du. \end{aligned}$$

Similarly, we get

2.11 $$\begin{aligned} {}_{k}^{s}J_{b^{-}}^{\alpha}F(a) =&\frac{ (b^{s+1}-a^{s+1} )^{\frac{\alpha}{k}}}{(s+1)^{\frac {\alpha}{k}}\Gamma_{k}(\alpha+k)}F(b) -\frac{(b-a)}{(s+1)^{\frac{\alpha}{k}}\Gamma_{k}(\alpha+k)} \\ &{}\times \int_{0}^{1} \bigl[\bigl(bu+(1-u)a \bigr)^{s+1}-a^{s+1} \bigr]^{\frac{\alpha }{k}}F'\bigl(bu+(1-u)a\bigr)\,du. \end{aligned}$$

Using the fact that $F(x)=f(x)+\tilde{f}(x)$ and by simple computation, from equalities (2.10) and (2.11), we get

2.12 $$\begin{aligned}& \frac{4(b^{s+1}-a^{s+1})^{\frac{\alpha}{k}}}{(b-a)} \biggl(\frac{f(a)+f(b)}{2} -\frac{(s+1)^{\frac{\alpha}{k}}\Gamma_{k}(\alpha +k)}{4(b^{s+1}-a^{s+1})^{\frac{\alpha}{k}}} \bigl[ {}_{k}^{s}J_{a^{+}}^{\alpha}F(b)+ {}_{k}^{s}J_{b^{-}}^{\alpha }F(a) \bigr] \biggr) \\& \quad = \int_{0}^{1} \bigl[ \bigl(\bigl(bu+(1-u)a \bigr)^{s+1}-a^{s+1} \bigr)^{\frac{\alpha}{k}} - \bigl(b^{s+1}-\bigl(bu+(1-u)a\bigr)^{s+1} \bigr)^{\frac{ \alpha}{k}} \bigr] \\& \qquad {}\times F'\bigl(bu+(1-u)a\bigr)\,du. \end{aligned}$$

Note that we have

$$F'\bigl(bu+(1-u)a\bigr)=f'\bigl(bu+(1-u)a \bigr)-f'\bigl(au+(1-u)b\bigr), \quad u\in[0,1]. $$

Then we can easily obtain

2.13 $$\begin{aligned}& \int_{0}^{1} \bigl(\bigl(bu+(1-u)a \bigr)^{s+1}-a^{s+1} \bigr)^{\frac{\alpha }{k}}F'\bigl(bu+(1-u)a\bigr)\,du \\& \quad = \int_{0}^{1} \bigl(\bigl(ta+(1-t)b \bigr)^{s+1}-a^{s+1} \bigr)^{\frac{\alpha }{k}}f'\bigl(ta+(1-t)b\bigr)\,dt \\& \qquad {}- \int_{0}^{1} \bigl(\bigl(bt+(1-t)a \bigr)^{s+1}-a^{s+1} \bigr)^{\frac{\alpha}{k}}f'\bigl(ta+(1-t)b\bigr)\,dt \end{aligned}$$

and

2.14 $$\begin{aligned}& \int_{0}^{1} \bigl(b^{s+1}-\bigl(bu+(1-u)a \bigr)^{s+1} \bigr)^{\frac{\alpha }{k}}F' \bigl(bu+(1-u)a\bigr)\,du \\& \quad = \int_{0}^{1} \bigl(b^{s+1}-\bigl(ta+(1-t)b \bigr)^{s+1} \bigr)^{\frac{\alpha }{k}}f' \bigl(ta+(1-t)b\bigr)\,dt \\& \qquad {}- \int_{0}^{1} \bigl(b^{s+1}-\bigl(bt+(1-t)a \bigr)^{s+1} \bigr)^{\frac{\alpha}{k}}f' \bigl(ta+(1-t)b\bigr)\,dt. \end{aligned}$$

Thus, the desired inequality (2.9) follows from inequalities (2.12), (2.13) and (2.14). □

For $\alpha,k>0$, we introduce the following operator:

$$\Im(s,x,y):= \int_{a}^{\frac{a+b}{2}}\vert x-u\vert\bigl\vert y^{s+1}-u^{s+1}\bigr\vert ^{\frac{\alpha}{k}}\,du - \int_{\frac{a+b}{2}}^{b}\vert x-u\vert\bigl\vert y^{s+1}-u^{s+1}\bigr\vert ^{\frac {\alpha}{k}}\,du, $$

$s\in\mathbb{R}\setminus\{-1\}$, $x,y\in[a,b]$.

Using Lemma 2.1, we can obtain the following $(k,s)$-fractional integral inequality.

### Theorem 2.2

*Let* $\alpha,k>0$ *and* $s\in\mathbb{R}\setminus\{-1\}$. *If* *f* *is a differentiable function on* $I^{\circ}$ *such that* $f'\in L[a,b]$ *with* $a< b$ *and* $\vert f' \vert$ *is convex on* $[a,b]$, *then*

2.15 $$\begin{aligned}& \biggl\vert \frac{f(a)+f(b)}{2}-\frac{(s+1)^{\frac{\alpha}{k}}\Gamma _{k}(\alpha+k)}{4(b^{s+1}-a^{s+1})^{\frac{\alpha}{k}}} \bigl[ {}_{k}^{s}J_{a^{+}}^{\alpha}F(b)+ {}_{k}^{s}J_{b^{-}}^{\alpha }F(a) \bigr]\biggr\vert \\& \quad \leq\frac{\Psi(s,\alpha,a,b)}{4(b^{s+1}-a^{s+1})^{\frac{\alpha }{k}}(b-a)}\bigl(\bigl\vert f'(a)\bigr\vert + \bigl\vert f'(b)\bigr\vert \bigr), \end{aligned}$$

*where*

$$\Psi(s,\alpha,a,b)=\Im(s,b,b)+\Im(s,a,b)-\Im(s,b,a)-\Im(s,a,a). $$

### Proof

Using Lemma 2.1 and the convexity of $\vert f'\vert$, we obtain

2.16 $$\begin{aligned}& \biggl\vert \frac{f(a)+f(b)}{2}-\frac{(s+1)^{\frac{\alpha}{k}}\Gamma _{k}(\alpha+k)}{4(b^{s+1}-a^{s+1})^{\frac{\alpha}{k}}} \bigl[ {}_{k}^{s}J_{a^{+}}^{\alpha}F(b)+ {}_{k}^{s}J_{b^{-}}^{\alpha }F(a) \bigr]\biggr\vert \\& \quad \leq \frac{(b-a)}{4(b^{s+1}-a^{s+1})^{\frac{\alpha}{k}}} \int_{0}^{1}\bigl\vert \nabla_{\alpha,s}(t) \bigr\vert \bigl\vert f'\bigl(ta+(1-t)b\bigr)\bigr\vert \,dt \\& \quad \leq \frac{(b-a)}{4(b^{s+1}-a^{s+1})^{\frac{\alpha}{k}}} \biggl(\bigl\vert f'(a)\bigr\vert \int_{0}^{1} t\bigl\vert \nabla_{\alpha,s}(t) \bigr\vert \,dt +\bigl\vert f'(b)\bigr\vert \int_{0}^{1} (1-t)\bigl\vert \nabla_{\alpha,s}(t) \bigr\vert \,dt \biggr). \end{aligned}$$

Note that

$$ \int_{0}^{1} t\bigl\vert \nabla_{\alpha,s}(t) \bigr\vert \,dt=\frac {1}{(b-a)^{2}} \int_{a}^{b}\bigl\vert \wp(u)\bigr\vert (b-u)\,du, $$

where

$$\begin{aligned} \wp(u) =&\bigl(u^{s+1}-a^{s+1}\bigr)^{\frac{\alpha }{k}}-\bigl((b+a-u)^{s+1}-a^{s+1}\bigr)^{\frac{\alpha}{k}} \\ &{}+\bigl(b^{s+1}-(b+a-u)^{s+1}\bigr)^{\frac{\alpha}{k}}-\bigl(b^{s+1}-u^{s+1}\bigr)^{\frac {\alpha}{k}}, \quad u\in[a,b]. \end{aligned}$$

Observe that ℘ is a non-decreasing function on $[a,b]$. Moreover, we have $\wp(a)=-2(b^{s+1}-a^{s+1})^{\frac{\alpha}{k}}<0$ and $\wp(\frac{a+b}{2})=0$. Thus, we have

$$\textstyle\begin{cases} \wp(u)\leq0 & \mbox{if } a\leq u\leq\frac{a+b}{2}, \\ \wp(u)>0 & \mbox{if } \frac{a+b}{2}< u\leq b. \end{cases} $$

So, we obtain

$$(b-a)^{2} \int_{0}^{1}t \bigl\vert \nabla_{\alpha,s}(t) \bigr\vert \,dt=\zeta _{1}+\zeta_{2}+\zeta_{3}+ \zeta_{4}, $$

where

$$\begin{aligned}& \zeta_{1} = \int_{a}^{\frac{a+b}{2}}(b-u) \bigl(b^{s+1}-u^{s+1} \bigr)^{\frac{\alpha}{k}}\,du - \int_{\frac{a+b}{2}}^{b} (b-u) \bigl(b^{s+1}-u^{s+1} \bigr)^{\frac{\alpha}{k}}\,du , \\& \zeta_{2} = - \int_{a}^{\frac{a+b}{2}}(b-u) \bigl(u^{s+1}-a^{s+1} \bigr)^{\frac{\alpha}{k}}\,du + \int_{\frac{a+b}{2}}^{b} (b-u) \bigl(u^{s+1}-a^{s+1} \bigr)^{\frac{\alpha}{k}}\,du, \\& \zeta_{3} = \int_{a}^{\frac{a+b}{2}}(b-u) \bigl((b+a-u)^{s+1}-a^{s+1} \bigr)^{\frac{\alpha}{k}}\,du - \int_{\frac{a+b}{2}}^{b} (b-u) \bigl((b+a-u)^{s+1}-a^{s+1} \bigr)^{\frac{\alpha}{k}}\,du, \\& \zeta_{4} = - \int_{a}^{\frac{a+b}{2}}(b-u) \bigl(b^{s+1}-(b+a-u)^{s+1} \bigr)^{\frac{\alpha}{k}}\,du + \int_{\frac{a+b}{2}}^{b} (b-u) \bigl(b^{s+1}-(b+a-u)^{s+1} \bigr)^{\frac{\alpha}{k}}\,du. \end{aligned}$$

Observe that $\zeta_{1}=\Im(s,b,b)$ and $\zeta_{2}=-\Im(s,b,a)$. Using the change of variable $v=a+b-u$, we get $\zeta_{3}=-\Im(s,a,a)$ and $\zeta_{4}=\Im(s,a,b)$. Thus, we obtain

2.17 $$ \int_{0}^{1}t \bigl\vert \nabla_{\alpha,s}(t) \bigr\vert \,dt=\frac{\Im (s,b,b)+\Im(s,a,b)-\Im(s,b,a)-\Im(s,a,a)}{(b-a)^{2}}. $$

Similarly,

2.18 $$ \int_{0}^{1}(1-t) \bigl\vert \nabla_{\alpha,s}(t) \bigr\vert \,dt=\frac{\Im (s,b,b)+\Im(s,a,b)-\Im(s,b,a)-\Im(s,a,a)}{(b-a)^{2}}. $$

So, the desired inequality (2.15) follows from inequalities (2.16), (2.17) and (2.18). □

## Conclusions

Lastly, we conclude this paper by remarking that we have obtained a Hermite-Hadamard inequality, an identity and a Hermite-Hadamard type inequality for a generalized *k*-fractional integral operator. Therefore, by suitably choosing the parameters, one can further easily obtain additional integral inequalities involving the various types of fractional integral operators from our main results.
